# Characterization of *Lactiplantibacillus paraplantarum* HK-1 and GABA Synthesis Under Simulated Gastrointestinal Conditions

**DOI:** 10.3390/foods14193345

**Published:** 2025-09-26

**Authors:** Susana Castro-Seriche, Joaquin Alvarez-Norambuena, Paulina Lincoñir-Campos, Cristian Gutiérrez-Zamorano, Alvaro Ruiz-Garrido, Bruno Jerez-Angulo, Apolinaria García-Cancino, Alonso Jerez-Morales

**Affiliations:** 1 Research and Development Department, Haiken Vetscience SpA Paicavi 483, Concepción 4070031, Chile; susana.castro@haikenlab.cl (S.C.-S.); paulina.linconir@haikenlab.cl (P.L.-C.); 2 Department of Veterinary Population Medicine, University of Minnesota, 1365 Gortner Ave, St. Paul, MN 55108, USA; jalvare@umn.edu; 3 Centro Interactivo de Ciencias, Artes y Tecnologías (CICAT), Universidad de Concepción, Avenida Cordillera 3582, Coronel 4190044, Chile; cgutierrezz@udec.cl; 4 Pathology and Preventive Medicine Department, School of Veterinary Sciences, Universidad de Concepción, Avenida Vicente Méndez 595, Chillan 3812120, Chile; aruiz@udec.cl; 5 School of Chemical Engineering, Pontificia Universidad Católica de Valparaíso, Brazil Avenue 2180, Valparaíso 2362854, Chile; bruno.jerez.a@mail.pucv.cl; 6 Laboratory of Bacterial Pathogenicity, Faculty of Biological Sciences, Universidad de Concepción, Chacabuco Avenue 1363, Concepción 4070386, Chile

**Keywords:** gamma-aminobutyric acid, *Lactiplantibacillus paraplantarum*, probiotic, glutamate decarboxylase, functional food, gastrointestinal simulation, gene expression, gut–brain axis

## Abstract

Gamma-aminobutyric acid (GABA) is a bioactive amino acid with anti-inflammatory and neurotransmitter properties, yet limited information exists regarding its production by *Lactiplantibacillus paraplantarum*. We evaluated factors that influence GABA synthesis by *L. paraplantarum* HK-1 and assessed its production in vitro and under simulated gastrointestinal conditions. GABA production was analyzed using HPLC with pre-column derivatization, gene expression was assessed through RT-qPCR, and probiotic characteristics were evaluated using standard microbiological methods. *L. paraplantarum* HK-1 demonstrated dose-dependent GABA production with monosodium glutamate (MSG) supplementation, achieving maximum levels at 500 mM MSG (161.1 µg/mL), which was significantly higher than those in other treatments (*p* < 0.01). A strong positive correlation was observed between MSG concentration and GABA production (r = 0.908, *p* = 0.002). Gene expression analysis revealed a 61.6-fold higher gadB expression at 500 mM MSG compared to 250 mM, though statistical significance with GABA production was not achieved (r = 0.741, *p* = 0.259). The strain exhibited appropriate probiotic characteristics including γ-hemolytic activity, bile salt tolerance, and acid resistance. Under simulated gastrointestinal conditions, maximum GABA production occurred in the distal colon (148.3 ± 19.0 µg/mL with probiotic vs. 7.2 ± 6.2 µg/mL control), with overall production significantly higher in probiotic-treated groups (*p* < 0.001). Overall, *L. paraplantarum* HK-1 produced GABA throughout gastrointestinal phases and showed traits consistent with probiotic use. These results position HK-1 as a promising GABA-producing candidate for functional food applications, pending in vivo validation.

## 1. Introduction

Gamma-aminobutyric acid (GABA) is a non-protein amino acid widely distributed among microorganisms, plants, and animals [1,2]. It is known as the primary inhibitory neurotransmitter in the central nervous system (CNS) of mammals, playing a key role in regulating neuronal excitability and maintaining synaptic balance [3,4,5]. In addition to its neurological function, recent studies have highlighted the anti-inflammatory potential of GABA at the gut level. In murine models, GABA reduced epithelial cell shedding, limit inflammatory cell infiltration, downregulate proinflammatory cytokines, and enhance anti-inflammatory responses in the intestinal mucosa [6,7,8]. GABA also has functional importance in other tissues such as gut, bladder, heart, lung, ovary, and pancreas [9]. In the colon, GABA-B receptors have been implicated in colonic visceral pain regulation, with positive allosteric modulators showing therapeutic potential for pain disorders [10]. In the bladder, both GABA-A and GABA-B receptors modulate contractility and micturition function, with GABA-A receptors involved in peripheral inhibition of bladder contractility and GABA-B receptors regulating overactive bladder conditions [10]. In pancreatic islets, functional GABA-A receptors with αβγ configuration are present in islet cells, where GABA serves as an important intraislet transmitter regulating β-cell secretion and function [11]. These effects suggest that GABA-producing microorganisms may serve as probiotic candidates for alleviating intestinal inflammatory disorders.

Besides its anti-inflammatory effect, GABA has been associated with several health benefits, such as improved sleep quality, reduction in mental and physical stress, enhanced immune response under stress conditions, delayed aging, vasodilation, and body weight modulation [12,13,14]. Consequently, there is increased interest in microbial sources of GABA, particularly among lactic acid bacteria (LAB), due to their “Generally Recognized As Safe” (GRAS) status, health-promoting effects, and ability to produce GABA through fermentation [9,15,16,17,18].

Several LAB species have demonstrated a capacity for high-yield GABA synthesis [19,20,21]. Among LAB species, *Lactiplantibacillus plantarum* strains demonstrate the highest reported GABA production levels, with some achieving up to 1419 μg/mL under optimized conditions [17], followed by Lactobacillus brevis strains reaching up to 847 mg/L [22], and *Lactobacillus buchneri* producing 235–445 μg/mL [23]. GABA biosynthesis in LAB is mediated primarily through the glutamate decarboxylase (GAD) system, in which the enzyme GAD decarboxylates L-glutamate into GABA with the release of CO_2_ [3,24]. This metabolic pathway is related to acid stress response mechanisms, as it consumes intracellular protons, thereby stabilizing pH homeostasis in acidic environments [25,26]. Genetic variability in the GAD system accounts for substantial difference in GABA production among LAB species and even between strains of the same species [27].

Environmental factors including temperature, pH, oxygen availability, and MSG concentration modulate GABA synthesis. MSG serves multiple roles: as direct substrate providing L-glutamate for GAD-mediated conversion to GABA, as a transcriptional regulator enhancing gadB gene expression, and as a pH buffering agent maintaining optimal conditions for enzymatic activity, making it often the rate-limiting factor in GABA biosynthesis [17,28,29]. While *L. plantarum* has been extensively studied for GABA production capacity, with numerous strains demonstrating variable yields ranging from 16.0 mg/kg to over 1400 μg/mL depending on culture conditions and substrate composition [17,30,31], research on *L. paraplantarum* remains limited, with investigations concentrating on antimicrobial activity through bacteriocin production [32], phosphorus absorption capacity for chronic kidney disease applications [33], and exopolysaccharide production [34], rather than GABA biosynthesis. Although the presence of glutamate decarboxylase genes in *L. paraplantarum* has been documented through phylogenetic analysis [35], no previous studies have experimentally demonstrated actual GABA production by this species. Notably, some strains of *L. plantarum* have been reported to synthesize GABA under optimized culture conditions, but studies focusing on *L. paraplantarum* remain limited [30,31]. Furthermore, while *L. plantarum* strains have been characterized for GABA production in various substrates and growing conditions [30], no previous studies have systematically evaluated the capacity of *L. paraplantarum* to maintain GABA synthesis under simulated gastrointestinal conditions, which is essential for determining the feasibility of in situ GABA production in the gut of the host and adds a translational dimension to probiotic functionality research.

## 2. Materials and Methods

### 2.1. Isolation and Characterization of GABA-Producing Strains

Fermented food products including traditional green cabbage, red cabbage, apples, and beetroot ferments of multiple origins were collected and processed for bacterial isolation. One gram of each sample was suspended in 9 mL of MRS broth (Condalab, Madrid, Spain) and incubated under microaerophilic conditions at 37 °C for 24–48 h. Aliquots were plated on MRS agar (Condalab, Madrid, Spain) supplemented with bromophenol blue (MRS-BPB) as a differential medium and incubated at 37 °C for 48 h. From these samples, 26 bacterial isolates were obtained and subjected to preliminary screening. Colonies were initially screened by Gram staining and tested for catalase and oxidase activity. Isolates that were Gram-positive, catalase-negative, and oxidase-negative isolates were selected for further characterization.

Genomic DNA was extracted using the Presto™ gDNA Bacteria Advanced Kit (Geneaid, Taiwan, China) following the manufacturer’s protocol. DNA concentration and purity were analyzed through spectrophotometry. Prior to genetic identification, all isolates were screened for GABA-producing potential using PCR amplification of the gadB gene. The core fragment of the gadB gene was amplified using chromosomal DNA as template with primers GAD1: 5′-TCGAGAAGCCGATCGCTTAGTTCG-3′ and GAD2: 5′-TATTGTCCGGTATAAGTGATGCCC-3′ [36], designed on highly conserved regions of the gadB sequence. Only gadB-positive isolates proceeded to genetic identification, which was carried out through 16S rRNA gene sequencing using the universal primers 27F (5′-AGAGTTTGATCMTGGCTCAG-3′) and 1492R (5′-TACGGYTACCTTGTTACGACTT-3′). Sequencing services were outsourced to Macrogen (Seoul, Republic of Korea).

### 2.2. Evaluation of GABA Production

#### 2.2.1. Culture Conditions

We evaluated MSG (Merck, Darmstadt, Germany) concentration effect on GABA production using five groups: (1) MRS broth alone (negative control), (2) MRS + *L. paraplantarum* HK-1 (bacterial control without MSG), (3) MRS + 10 g/L glucose + 250 mM MSG without bacteria (sub-strate control), (4) MRS + 10 g/L glucose + 250 mM MSG + *L. paraplantarum* HK-1 (low MSG treatment), and (5) MRS + 10 g/L glucose + 500 mM MSG + *L. paraplantarum* HK-1 (high MSG treatment). Glucose (10 g/L) was added specifically to MSG-supplemented treatments based on previous studies showing that glucose concentration positively correlates with GABA yield and cell density [37]. The bacterial inoculation rate was 1% *v*/*v* (10^8^ CFU/mL initial concentration). Cultures (duplicate) were adjusted to pH 6.0 and incubated at 37 °C for 48 and 96 h. GABA quantification and gene expression analysis were performed at 48 h, while pH monitoring and viable cell counts were continued until 96 h to assess culture stability and long-term viability.

#### 2.2.2. Gene Expression Analysis Through RT-qPCR and Method Validation

Expression of the *gadB* and 16S rRNA genes was assessed using real-time reverse transcription quantitative PCR (Leia-x4, Biobase, Jinan, China) using the following primers for *gadB* GDLp-F (5′-AACCAGTCTTTGGTGCGCCT-3′)/GDLp-R (5′-AACGGCTTCGGGTTCCATAT-3′); and 16S rRNA Lac16sF (5′-AGCAGTAGGGAATCTTCCA-3′)/Lac16sR (5′-CACCGCTACACATGGAG-3′). Total RNA was extracted from 48 h cultures using the OMEGA E.Z.N.A.^®^ Total RNA Kit (Omega Bio-Tek, Norcross, GA, USA), with DNase I treatment (New England BioLabs, Ipswich, MA, USA) to remove genomic DNA, following the manufacturers’ protocol. Gene expression analysis was performed with biological replicates per group, representing preliminary data that requires validation with larger sample sizes for definitive conclusions. Relative expression was calculated using the 2^−ΔΔCt^ method with 16S rRNA as the reference gene and group C2 as the calibrator [38]. Method validation included generation of standard curves from serial dilutions of *L. paraplantarum* HK-1 genomic DNA. DNA concentration was quantified using an Epoch™ spectrophotometer (BioTek, Winooski, VT, USA), and serial ten-fold dilutions were prepared from the initial genomic DNA stock. Amplification efficiency (acceptable range: 90–110%) and linearity (R^2^ > 0.98) were verified for each gene. Reverse transcription and amplification were performed using the Brilliant II Ultra-Fast SYBR^®^ Green QRT-PCR Master Mix 1-Step Kit (Agilent, Santa Clara, CA, USA).

#### 2.2.3. GABA Quantification by HPLC

GABA levels were determined using high-performance liquid chromatography (HPLC) (EClassical^®^ 3100 System, Dalian Elite Analytical Instruments Co., Dalian, China) with pre-column derivatization. A standard GABA solution (Sigma-Aldrich, St. Louis, MO, USA) was prepared at 1.84 mg/mL and serially ten-fold diluted.

Fermentation broth samples were centrifuged at 3000× *g* for 5 min and further filtered through 0.22 μm membranes prior to derivatization. Derivatization was carried out by mixing 100 μL of sample (or standard) with 900 μL of 0.1 M sodium bicarbonate buffer (pH 8.7) and 1000 μL of derivatization solution, prepared by dissolving 5 mg of dansyl chloride (Sigma-Aldrich, St. Louis, MO, USA) in 10 mL of acetone. The solution was vigorously homogenized for 30 s and incubated in the dark at 55 °C for 1 h. After incubation, samples were cooled to room temperature and filtered again (0.22 μm) prior to chromatographic analysis [3,25].

HPLC was conducted using an EClassical 3100 System equipped with a UV detector (Dalian Elite Analytical Instruments Co., Dalian, China) and a NH2 column (4.6 × 250 mm, 5 μm, Intersil, GL Sciences, Torrance, CA, USA) maintained at 25 °C. The mobile phase consisted of two solutions (A) 50 mM sodium acetate, methanol, and tetrahydrofuran in a ratio of 5:75:420 *v*/*v*/*v*; and (B) 100% methanol. Separation was achieved using a linear gradient elution from 1% to 100% phase B at a flow rate of 1.0 mL/min. Detection was performed at 254 nm with an injection volume of 20 μL. The retention time of the derivatized GABA was approximately 21 min.

Method validation involved the assessment of linearity by regression analysis, as well as determination of the limit of detection (LOD) and limit of quantification (LOQ). GABA quantification in samples was performed by interpolating peak areas against the calibration curve using Kromstation software version is 3.0.0.148. (Dalian Elite Analytical Instruments Co., Dalian, China) [28].

### 2.3. Safety and Probiotic Assessment

#### 2.3.1. Antimicrobial Susceptibility Testing

Bacterial suspension of *L. paraplantarum* HK-1 were adjusted to McFarland 0.5 standard (1.5 × 10^8^ CFU/mL) and spread onto MRS agar plates (Condalab, Madrid, Spain). Antibiotic susceptibility was assessed using the disk diffusion method. Plates were incubated at 37 °C for 48 h, and the diameters of the inhibition halos were measured. Susceptibility was evaluated against to the following antibiotics: penicillin, sulfa-trimethoprim, tetracycline, kanamycin, ceftriaxone, ampicillin, erythromycin, amoxicillin–clavulanic acid and amikacin (Mastdiscs, Bootle, UK).

#### 2.3.2. Hemolytic Activity Assay

Hemolysis was evaluated on MRS agar (Condalab, Madrid, Spain) supplemented with 5% swine blood. Plates were incubated at 37 °C for 48 h and the presence of α, β, or γ hemolysis around colonies was assessed.

#### 2.3.3. Bile Salt Tolerance Testing

Tolerance to bile salts was assessed in MRS broth supplemented with 0.5%, 1%, and 1.5% Ox-Gall (Merck, Darmstadt, Germany). Cultures were adjusted to a McFarland 2 standard (6.0 × 10^8^ CFU/mL) and incubated at 37 °C. Viable cell counts were carried out at 0, 1, 2, and 3 h using the microdrop plating method.

#### 2.3.4. Acid Resistance Evaluation

Tolerance to acidic conditions was evaluated in MRS broth (Condalab, Madrid, Spain) adjusted to pH 2.0, 2.5, and 3.0 using 10 M HCl. Cultures were standardized to McFarland 2 (6.0 × 10^8^ CFU/mL) and incubated at 37 °C. Viable cell counts were carried out at 0, 1, 2, and 3 h to assess survivability.

#### 2.3.5. Antimicrobial Activity Assessment

Antimicrobial activity was evaluated using the well diffusion method against *E. coli* ATCC 8739, *S. Typhimurium* ATCC 14028, *Pseudomonas aeruginosa* ATCC 9027, and *Staphylococcus aureus* ATCC 29213 (Thermo Fisher Scientific, USA). Pathogenic bacterial cultures were adjusted to 0.5 McFarland standard (≅1.5 × 10^8^ CFU/mL) following established agar well diffusion protocols for antimicrobial evaluation [39]. Three fractions were prepared: whole culture (WC), supernatant (S), and bacterial pellet (P). Wells of 6 mm diameter were made in trypticase soy agar plates (Condalab, Madrid, Spain) previously seeded with the pathogenic strains, and 100 μL of each fraction was added to the wells. Plates were incubated at 37 °C for 24 h, and inhibition halos were measured. Inhibition zones were measured (mm) after 48 h of incubation under appropriate conditions. Results were categorized following the evaluation system described by Yerlikaya et al. [40], halos of 9 mm or less were considered as resistant (negative), halos of 10–15 mm as moderate sensitivity (+), halos of 16–19 mm as intermediate sensitivity (++), and halos of 20 mm or more as high sensitivity (+++).

### 2.4. Gastrointestinal Simulation Protocol

Simulated gastrointestinal tract was evaluated using three sequential phases: gastric, intestinal, and colonic, following a modified protocol [41]. Although such models are used to study probiotics, they cannot fully reproduce in vivo physiology. Key limitations include the absence of host immune response, resident microbiome interactions, peristaltic movements, and dynamic nutrient and metabolite turnover [42]. As a results from these limitations, the results are interpreted as indicative trends rather than direct predictor of in vivo probiotic performance.

#### 2.4.1. Gastric Phase Simulation

Simulated gastric fluid consisted of 200 mL of autoclaved distilled water (121 °C, 15 min) enriched with macronutrients carbohydrates 59.12% (50% sugar and 50% wheat flour), lipids 28.26% (commercial sunflower oil), proteins 12.62% (meat peptone, Sigma-Aldrich, St. Louis, MO, USA) and the pH was adjusted to 3.0 to simulate fed-state gastric conditions [41]. The mixture was supplemented with 0.33 g pepsin and 0.086 g amylase (Sigma-Aldrich, St. Louis, MO, USA), then inoculated with 1 mL of *L. paraplantarum* HK-1 culture (10^9^ CFU/mL, 48 h in MRS broth). A parallel control group was processed identically through all three phases (gastric, intestinal, and colonic) using the same medium composition and conditions, but without bacterial inoculation. Instead of the bacterial culture, 1 mL of sterile MRS broth was added to maintain equivalent volumes and conditions between experimental and control groups. Incubation was conducted at 37 °C, with orbital agitation at 180 rpm for 2 h. Samples were collected at 0 and 2 h for pH measurement and viable cell counts.

#### 2.4.2. Small Intestine Phase Simulation

Following the gastric phase, the pH was adjusted to 5.0 using 10 M NaOH (Merck, Darmstadt, Germany), and the media was supplemented with 0.3% bile salts NaOH (Merck, Darmstadt, Germany) and pancreatic enzymes (6500 FIP lipase, 5500 FIP amylase, 400 FIP protease units). Incubation was carried out for 4 h at 37 °C with orbital agitation at 180 rpm. Samples were taken at 0 and 4 h for pH and viable cell counts. An additional sample was taken at 2 h for pH measurement and adjusted if needed.

#### 2.4.3. Colonic Phase Simulation

At the end of the small intestinal phase, the pH was adjusted to 5.5–6.1 and maintained throughout the duration of the colonic phase. The culture was incubated for 66 h at 37 °C with orbital agitation at 180 rpm. Samples were collected every 22 h (0, 22, 44, and 66 h) for pH and viable cell counts.

#### 2.4.4. Viability and GABA Quantification

Viable cell counts were determined by preparing serial dilutions in PBS up to 10^−8^ and plating 20 μL in triplicate on MRS agar (Condalab, Madrid, Spain) using the microdrop technique. Plates were incubated at 37 °C for 48 h prior to viable cell count. At each phase of the simulated gastrointestinal process, samples were also collected for GABA quantification using the HPLC method described in Section 2.2.3. Control group samples were processed identically for GABA quantification, with viable cell counts expected to be zero due to the absence of bacterial inoculation.

### 2.5. Statistical Analysis

Statistical differences in GABA production among groups were evaluated using the Kruskal–Wallis test followed by Dunn’s post hoc test with Benjamini–Hochberg correction for multiple comparisons. Pearson correlation analysis was used to assess the relationships between variables. All analyses were performed in R version 4.4.2, with significance set at *p* < 0.05.

## 3. Results

### 3.1. Identification and Characterization of Lactiplantibacillus paraplantarum HK-1

16S rRNA gene sequencing identified the strain as *L. paraplantarum* HK-1, isolated from fermented beetroot, showing 100% sequence identity with *L. paraplantarum* type strain (Accession number: NR_025447.1), confirming its classification as a lactic acid bacterium within the genus *Lactiplantibacillus*.

### 3.2. Factors Influencing GABA Production

#### 3.2.1. Effect of Glutamate Concentration on GABA Production

Prior to GABA quantification, the HPLC method was validated according to ICH guidelines. The method demonstrated excellent linearity (R^2^ = 0.9941) across the concentration range of 6.13–1533.33 µg/mL with acceptable precision and sensitivity (Table 1). *L. paraplantarum* HK-1 demonstrated a dose-dependent increase in GABA production capacity that increased with MSG supplementation.

**Figure 1:**
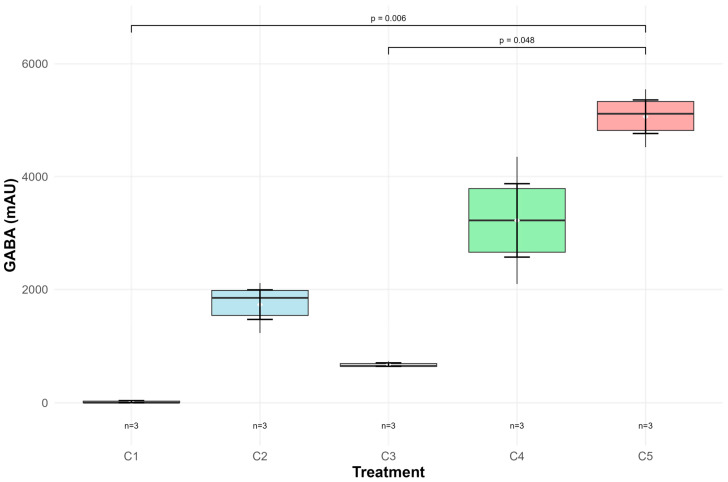
GABA synthesis by *L. paraplantarum* HK-1 under different MSG supplementation conditions.

GABA production differed significantly among groups (*p* < 0.001; Figure 1). Group C5 (500 mM MSG) achieved highest production (161.1 µg/mL), followed by C4 (102.3 µg/mL) and C2 (54.6 µg/mL). Control groups C1 and C3 exhibited GABA concentrations below the limit of detection (LOD, 24.90 µg/mL), with no detectable values and 20.7 ± 0.9 µg/mL, respectively.

Pearson correlation analysis demonstrated a strong positive relationship between MSG concentration and GABA production (r = 0.908; *p* = 0.002; Table 2), confirming the substrate-dependent nature of GABA biosynthesis and suggesting that higher MSG concentrations may be required to achieve statistically significant increases in GABA production by this strain.

#### 3.2.2. Effect of pH on GABA Production

**Figure 2:**
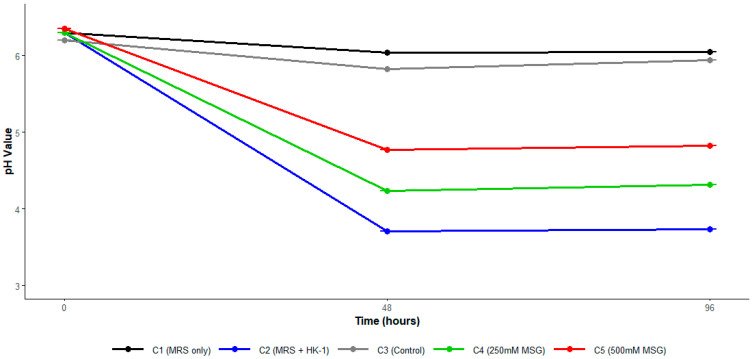
pH variation across treatment groups during *L. paraplantarum* HK-1 fermentation.

No statistically significant differences were observed across experimental groups (*p* = 0.082; Figure 2), distinct patterns in pH evolution were evident during the 96 h fermentation period. Control groups C1 and C3 maintained relatively stable pH values throughout the experiment (C1: 6.30, 6.04, 6.05; C3: 6.20, 5.82, 5.94), while bacterial cultures exhibited pronounced acidification. Group C2 showed the greatest pH de-crease (6.30, 3.71, 3.73), followed by C4 (6.30, 4.23, 4.31) and C5 (6.35, 4.77, 4.82), Notably, higher MSG concentrations appeared to moderate acidification, with C5 exhibiting less pH decline than C2 and C4, suggesting a potential buffering effect. A strong positive correlation was observed between pH and GABA production (r = 0.909, *p* = 0.002; Table 2), pH negatively correlated with viable cell count (r = −0.118, *p* = 0.78; Table 2, suggesting that acidification may affect cell viability.

#### 3.2.3. Relationship Between Viability and GABA Production

**Figure 3:**
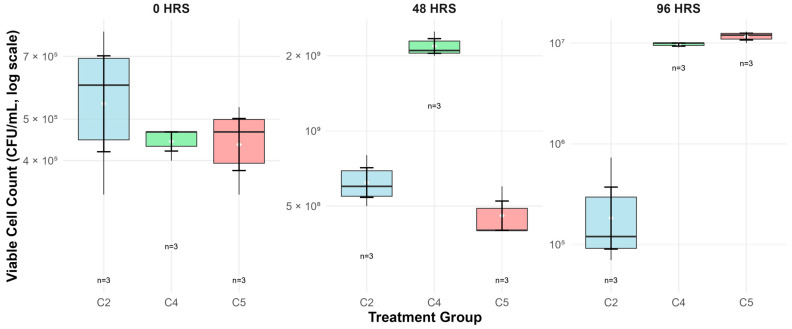
Viable cells count of *L. paraplantarum* HK-1 at 0, 48 and 96 h per group.

Viable cell counts exhibited dramatic growth patterns followed by severe population decline, though no statistically significant differences were observed between groups at individual time points (Figure 3). Starting from similar initial densities, all bacterial cultures experienced massive population expansion by 48 h.

Group C4 achieved the highest peak viability at 48 h (2.20 × 10^9^ ± 2.65 × 10^8^ CFU/mL), representing an extraordinary 4950-fold increase from initial counts. Groups C2 and C5 showed more moderate but still substantial growth, reaching 6.33 × 10^8^ ± 1.53 × 10^8^ CFU/mL (1096-fold increase) and 4.67 × 10^8^ ± 1.15 × 10^8^ CFU/mL (1050-fold increase), respectively.

By 96 h, all cultures experienced population decline, with survival rates of 0.05% (C2: 3.07 × 10^5^ ± 3.67 × 10^5^ CFU/mL), 0.44% (C4: 9.67 × 10^6^ ± 5.77 × 10^5^ CFU/mL), and 2.50% (C5: 1.17 × 10^7^ ± 1.53 × 10^6^ CFU/mL) relative to 48 h peaks. Notably, C5 exhibited greater survival despite lower peak counts, suggesting that higher MSG concentrations may enhance cellular stress tolerance during extended fermentation. No significant correlation was observed between CFU/mL and GABA production at 48 h (r = −0.132, *p* = 0.755; Table 2), indicating that GABA synthesis is not directly proportional to bio-mass density.

#### 3.2.4. Expression of *gadB* and Association with GABA Production

**Figure 4:**
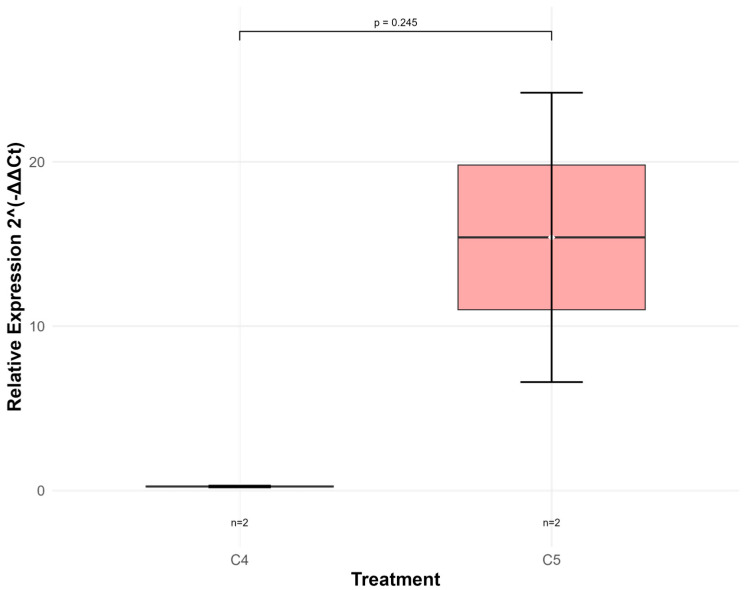
Gene expression of *gadB* gene at 48 h of fermentation by *L. paraplantarum* HK-1 under different glutamate concentrations.

Expression analysis of the *gadB* gene was conducted in groups C2, C4 and C5, as control groups C1 and C3 showed no bacterial growth. Group C2 served as the calibrator for relative expression analysis. Despite the limited sample size (n = 2 per group), these preliminary results suggest a potential functional link between gene expression and metabolite synthesis that requires further validation with larger sample sizes to achieve statistical significance. (Figure 4).

Group C5 (500 mM MSG) exhibited marked upregulation *gadB* expression (15.4 ± 12.4) compared to C4 (250 mM MSG: 0.25 ± 0.071), representing a 61.6-fold difference.

Despite the limited sample size (n = 2 per group), a positive trend was observed between *gadB* relative expression and GABA production, though statistical significance could not be established (r = 0.741, *p* = 0.259; Table 2). This suggests a potential functional link between gene expression and metabolite synthesis that requires further validation with larger sample sizes. Additionally, *gadB* expression showed a positive correlation with MSG concentration (r = 0.773, *p* = 0.227; Table 2), indicating that glutamate availability may influence transcriptional regulation of the glutamate decarboxylase system.

The substantial upregulation of *gadB* in C5 corresponded with the highest GABA concentrations observed (161.1 µg/mL), while the downregulated expression in C4 aligned with intermediate GABA production (102.3 µg/mL). These findings suggest that high MSG concentrations (500 mM) enhance GABA biosynthesis through transcriptional activation of the glutamate decarboxylase system, establishing a clear substrate-gene-product relationship in *L. paraplantarum* HK-1.

### 3.3. Safety and Probiotic Potential Evaluation

#### 3.3.1. Antimicrobial Susceptibility

In Table 3, we observed that the antimicrobial susceptibility of *L. paraplantarum* HK-1 was consistent with several clinically relevant antibiotics, including penicillin, tetracycline, ampicillin, erythromycin, and multiple β-lactams (ceftiofur, cefadroxil, amoxicillin–clavulanic acid). Resistance was observed to aminoglycosides such as kanamycin and amikacin, as well as to vancomycin and gentamicin, an expected trait among lactobacilli and considered intrinsic. Intermediate sensitivity was noted for sulfa-trimethoprim and ciprofloxacin. This susceptibility profile supports the safety of this strain for potential probiotic use.

#### 3.3.2. Hemolytic Activity

After 48 h of incubation on MRS agar supplemented with 5% swine blood, *L. paraplantarum* HK-1 displayed no visible hemolysis, confirming a γ-hemolytic phenotype. The absence of hemolytic activity further supports its safety profile for probiotic applications.

#### 3.3.3. Bile Salt Tolerance

The strain exhibited tolerance to bile salts at all tested concentrations. After 3 h of exposure, the reductions in viability were 56% (0.5%), 21% (1.0%), and 51% (1.5%). In 0.5% and 1.0% treatments, viable cell counts remained within the same order of magnitude, indicating good adaptation to physiological bile salt levels typically encountered in the small intestine.

#### 3.3.4. Acidic pH Tolerance

*L. paraplantarum* HK-1 maintained viability after 3 h of exposure at pH 2.5 and 3.0, with reductions in cell count of 22% and 12%, respectively. At pH 2.0, viability was lost after 2 h. Despite this limitation, the strain exhibited sufficient acid tolerance to survive typical gastric conditions.

#### 3.3.5. Antimicrobial Activity

*L. paraplantarum* HK-1 demonstrated moderate antimicrobial activity against the tested pathogenic strains. The whole culture fraction exhibited inhibition zones of 14 mm against *E. coli* ATCC 8739 and *S. aureus* ATCC 29213, and 12 mm against *P. aeruginosa* ATCC 9027 and *S. Typhimurium* ATCC 14028, corresponding to moderate sensitivity.

The supernatant fraction showed identical antimicrobial performance, producing inhibition halos of 14 mm against *E. coli* and *S. aureus*, and 12 mm against *P. aeruginosa* and *S. Typhimurium* (moderate sensitivity, +). In contrast, the bacterial pellet fraction displayed no antimicrobial activity, with inhibition zones of 6 mm (resistance, −) against all tested pathogens.

### 3.4. Simulated Gastrointestinal Conditions

*L. paraplantarum* HK-1 demonstrated progressive GABA production throughout the simulated gastrointestinal transit, with significant differences between probiotic-supplemented and control groups, particularly in the final colonic phase. GABA concentrations were measured across four sequential sections: Small Intestine-1, Small Intestine-2, Colon-1, and Colon-2 (Table 4), comparing treatments with and without probiotic supplementation.

In the small intestinal phases, GABA levels remained below the analytical method’s limit of detection in both groups. Small Intestine-1 showed minimal concentrations of 10.5 ± 6.8 μg/mL with probiotics versus non-detectable levels (−0.4 ± 0.1 μg/mL) in controls, while Small Intestine-2 demonstrated 15.7 ± 12.1 μg/mL versus 6.7 ± 4.6 μg/mL, respectively.

The colonic phases revealed a dramatic probiotic effect, particularly in the final section. Colon-1 showed modest concentrations below the LOD, with 19.0 ± 4.2 μg/mL in probiotic treatment compared to 9.8 ± 6.8 μg/mL in controls. Most remarkably, Colon-2 exhibited the only quantifiable GABA concentrations throughout the entire simulation, reaching 148.3 ± 19.0 μg/mL with probiotic supplementation compared to 7.2 ± 6.2 μg/mL in controls, representing a striking 20-fold increase.

**Figure 5:**
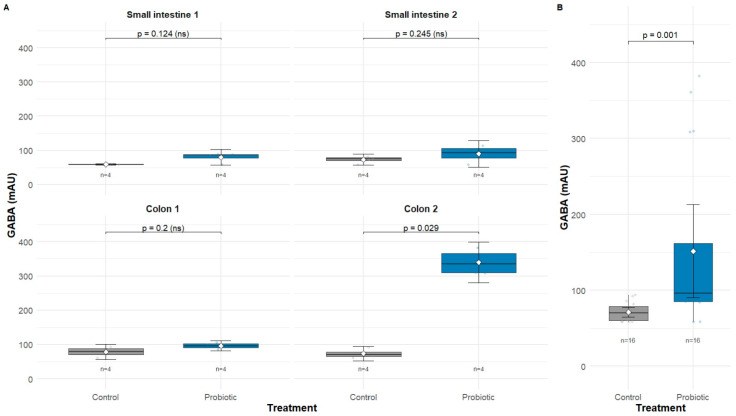
Effect of *L. paraplantarum* HK-1 on GABA production ex vivo gastrointestinal simulation. (**A**) GABA concentrations (µg/mL) throughout all simulate gastrointestinal sections, (**B**) overall GABA production in gastrointestinal simulation (µg/mL). ns means no significant difference.

Although differences within individual section were not significant (*p* > 0.05; Figure 5A), overall GABA across all phases was higher with HK-1 than controls when considering all gastrointestinal sections combined (*p* < 0.001; Figure 5B). A significant negative correlation was observed between viable cell count and GABA concentration (r = −0.447, *p* = 0.028). This pattern suggests a potential stress-adaptive response but should be interpreted cautiously as multiple (i.e., loss of viability in gastric and intestinal phases or colon-specific conditions) factors may contribute.

## 4. Discussion

We investigated factors that modulate GABA production by *L. paraplantarum* HK-1 and tested synthesis under simulated gastrointestinal conditions. Findings support HK-1 as a GABA-producing strain with probiotic potential, pending in vivo validation.

Analysis of factors influencing GABA production revealed significant positive correlations. MSG availability showed a strong positive correlation with GABA pro-duction (r = 0.908, *p* = 0.002), confirming its role as a determining substrate in biosynthesis. Maximum production (161.1 µg/mL) was achieved with 500 mM MSG after 48 h of cultivation. These results are consistent with previous studies, such as that by Diez-Gutiérrez [17], who optimized GABA production using *L. plantarum* K16 over 96 h, observing a significant increase in production from 174.17 μg/mL with 100 mM MSG to 1419.93 μg/mL with 500 mM MSG, followed by a reduction to 1027.81 μg/mL with 550 mM MSG. These concentration-dependent patterns align with those reported in other strains by other authors [17,31,43], suggesting a conserved regulatory mechanism of GABA synthesis by substrate availability.

Medium pH also emerged as a regulatory factor, with correlation analysis showing a strong positive relationship with GABA production (r = 0.909, *p* = 0.002), indicating that pH values below 4 may decrease GABA production and values near 5 seem optimal. Additionally, a negative correlation was observed between pH and bacterial count, though this was not statistically significant (r = −0.118, *p* = 0.78). Diez-Gutiérrez [17] evaluated the effect of initial pH on GABA production by L. plantarum K16 strain, finding that at pH 4.0 production was low (197.5 μg/mL), increasing significantly to 951.05 μg/mL at pH 4.5, and reaching its maximum at pH 5.5 (1419.93 μg/mL). This suggests that overly acidic conditions could inhibit GAD enzymatic activity and re-duce GABA synthesis in specific strains. Published studies have established that the optimal pH for GABA biosynthesis in various lactic acid bacteria is around 5.0. This optimal pH value has been independently confirmed in multiple species, including L. brevis NCL912 strain [22] and *L. buchneri* strains [23]. Similarly, Cai et al. [43] evaluated the impact of initial pH on *L. plantarum* FRT7, observing that the highest pH values within those evaluated favor GABA production, reaching 335.88 ± 9.19 μg/mL at pH 7.0. Conversely, in other bacterial genera, it has been demonstrated that GABA production under acidic pH conditions is significantly higher than at pH values near neutrality. Otaru [44] observed that in *Bacteroides thetaiotaomicron* DSM 2079, GABA production increases considerably as pH decreases, reaching maximum levels at pH 3.1 (3.78 ± 0.25 mM in minimal medium with glutamine and 6.62 ± 0.68 mM in minimal medium with glutamate). This highlights that optimal conditions for GABA synthesis are highly strain-dependent [30].

An interesting effect of the potential industrial use for exploiting this gene is the pH modulating effect exerted by MSG. Medium pH emerged as a regulatory factor for viability during fermentation, revealing a clear pattern in pH regulation at both 48 and 96 h (Figure 2). Interestingly, higher MSG concentrations appeared to moderate acidification, with C5 showing less pH reduction than C2 and C4, suggesting a potential buffering effect. It has been reported that this sustained buffering effect of MSG could simplify and make more efficient bioprocesses involving microorganisms expressing the *gad* gene [29], potentially reducing the need for external pH control systems and, consequently, the operational costs associated with maintaining optimal fermentation conditions.

The GAD system is widely distributed among lactic acid bacteria and represents the main pathway for GABA production [15]. This system in *L. paraplantarum* HK-1 comprises the GadB enzyme, which catalyzes the irreversible decarboxylation of L-glutamate using pyridoxal-5′-phosphate (PLP) as a cofactor and consuming H+ in the process, resulting in GABA formation and CO_2_ release. The *gadB* gene is co-transcribed in tandem with *gadC*, forming a coordinated functional unit that enables an efficient GAD system response [28]. This gene organization facilitates the proton-consuming decarboxylation reaction to contribute directly to intracellular pH homeostasis, providing the bacterium with an acid resistance mechanism and adaptive advantage in adverse environments such as the gastrointestinal tract [34]. Our results demonstrate that GABA production in *L. paraplantarum* HK-1 is governed by a complex regulatory network, with substrate availability playing a fundamental role. Maximum production (161.1 µg/mL) was achieved with 500 mM MSG after 48 h of cultivation, showing a strong correlation between MSG concentration and GABA production (r = 0.908, *p* = 0.002). This response to substrate availability is reflected at the transcriptional level, where we observed maximum relative expression of the *gadB* gene (15.4 ± 12.4) under the same condition, though statistical significance between gene expression and GABA production was not achieved (r = 0.741, *p* = 0.259). Although the correlation between *gadB* and pH was not significant, several studies have demonstrated that pH is an important regulatory factor in *gadB* transcription and enzymatic activity [28,45,46]. It is important to highlight that the mere presence of the *gadB* gene does not guarantee GABA production, as demonstrated in *L. fermentum* strains that possess the gene but lack the capacity to produce GABA [47].

The gastrointestinal simulation suggests that *L. paraplantarum* HK-1 be relevant for probiotic applications. However, these findings should be interpreted cautiously, until validated in vivo. Despite declining cell viability during the gastrointestinal simulation, GABA) remain detectable and increased in later phases. GABA and viable cell counts were negatively correlated (r = −0.447, *p* = 0.028), consistent with stress-induced activation of the GAD system or greater extracellular accumulation when biomass is lower [44,48,49]. However, the exact mechanism remains unclear and requires further mechanistic investigation. Studies demonstrate that GABA production peaks during late exponential phase when cells face environmental stress rather than during healthy growth conditions [44]. However, other studies have reported that higher cell biomass increases GABA production [50]. Li et al. [51], found that higher inoculum concentration can produce more cell biomass, potentially allowing greater glutamate to GABA conversion, while recent studies have shown that GABA yield and cell density increased as glucose concentration increased [37]. This apparent contradiction suggests that the relationship between cell viability and GABA production may be strain-dependent and influenced by, specific culture conditions, and fermentation timing, since those studies established optimized conditions for microorganisms, while this simulation evaluated strain effectiveness in an ex vivo system that replicates adverse gastrointestinal tract conditions.

GABA production negatively correlated with cell viability during gastrointestinal simulation. Cell loss occurred mainly during gastric and intestinal phases due to acidic pH, enzymes, and bile salts. However, maximum GABA production occurred in the colonic phase despite reduced viable populations. This suggests that specific simulated colon conditions provide a favorable environment for expression of genes related to GABA synthesis, where surviving cells compensate for population reduction through increased specific metabolic activity. Previous studies have documented similar patterns in other experimental models. For example, Casertano et al. [52] employed a SHIME system to evaluate GABA production in different gastrointestinal tract regions. Their findings indicated that GABA was generated predominantly in the ascending, transverse, and descending colon, correlating with pH values of 5.6, 6.15, and 6.6, respectively. Collectively, these results underscore the importance of the colon as a key site for GABA production by probiotics, which could be leveraged in therapies targeting disorders associated with GABAergic neurotransmission.

The capacity of *L. paraplantarum* HK-1 to produce GABA under simulated gastrointestinal conditions suggests its potential as a biotherapeutic tool, particularly considering that GABA levels are diminished in multiple inflammatory diseases. Clinical studies have documented GABA deficits in pathologies such as multiple sclerosis, ischemic stroke, and ulcerative colitis [53], while in irritable bowel syndrome with diarrhea predominance (IBS-D), a complex dysregulation of the GABAergic system is observed, characterized by reduced levels of GABA, GAD2, and GABAB receptors, along with increased expression of the GAT-2 transporter This dysregulation has been directly associated with visceral pain, a cardinal symptom of IBS, suggesting that GABA-producing probiotics such as *L. paraplantarum* HK-1 warrant further investigation for potential therapeutic applications, though extensive in vivo validation remains essential.

This study has several limitations that should be acknowledged. First, the gene expression analysis was limited by a small sample size (n = 2 per group), requiring further validation with larger sample sizes to confirm the relationship between *gadB* expression and GABA production. Second, the in vitro gastrointestinal simulation, while methodologically rigorous, cannot fully replicate the complexity of the in vivo intestinal environment, including interactions with resident microbiota, individual physiological variations, and host immune factors. Finally, in vivo validation studies are needed to confirm the probiotic functionality and GABA production capacity of *L. paraplantarum* HK-1 under actual physiological conditions.

## 5. Conclusions

*L. paraplantarum* HK-1 exhibited appropriate probiotic safety characteristics and maintained GABA production throughout simulated gastrointestinal conditions, achieving maximum synthesis in the distal colon (148.3 µg/mL vs. 7.2 µg/mL control, *p* < 0.001). Several limitations must be acknowledged: the in vitro simulation cannot replicate in vivo complexity, gene expression analysis requires larger sample sizes, and probiotic functionality needs validation through animal studies and clinical trials. Future research should focus on in vivo validation, clinical safety and efficacy studies, mechanistic investigations of viability-GABA relationships, and fermentation optimization for industrial applications.

## Figures and Tables

**Table 1:** Standard curve parameters and method validation for GABA quantification by HPLC.

Parameter	Value
Linearity Range (µg/mL)	6.13–1533.33
Regression Equation	y = 32.6x − 525.85
R^2^	0.9941
LOD (µg/mL)	24.90
LOQ (µg/mL)	75.60

**Table 2:** Correlation matrix of fermentation parameters and GABA production by *L. paraplantarum* HK-1.

Sample 1	Sample 2	N	Correlation	95% IC	*p*-Value
MSG (mM)	GABA (mAU)	8	0.908	(0.566; 0.983)	0.002
pH_48 h	GABA (mAU)	8	0.909	(0.567; 0.984)	0.002
UFC_48 h	GABA (mAU)	8	−0.132	(−0.766; 0.631)	0.755
gadB_RE	GABA (mAU)	4	0.741	(−0.765; 0.994)	0.259
MSG (mM)	gadB_RE	4	0.773	(−0.732; 0.995)	0.227
pH_48 h	UFC_48 h	8	−0.118	−0.76	0.78

UFC/mL = Colony Forming Units per milliliter; RE = Relative Expression of gadB gene; 95% IC = 95% Confidence Interval.

**Table 3:** Antibiotic susceptibility testing of *L. paraplantarum* HK-1 by disk diffusion method.

Antibiotic	Concentration (μg)	Halo Diameter (mm)	Interpretation
Penicillin	10	30	Susceptible
Sulfa Trimetoprim	23.75/1.25	15	Intermediate
Tetracycline	30	23	Susceptible
Kanamycin	30	6	Resistant
Ceftriaxone	30	30	Susceptible
Ampicillin	10	30	Susceptible
Erythromycin	15	30	Susceptible
Amoxicillin/Clavulanic Acid	20-11	30	Susceptible
Amikacin	30	6	Resistant
Gentamicin	10	6	Resistant
Vancomycin	30	6	Resistant
Ciprofloxacin	5	15	Intermediate

**Table 4:** Viability, pH, and GABA production during the simulated gastrointestinal passage of *L. paraplantarum* HK-1.

Phase	Cumulative Time (h)	pH	Count (CFU/mL)	Survival (%)	GABA (µg/mL)
Stomach	0	3.50	4.67 × 10^8^ ± 2.89 × 10^7^	100	ND
Stomach	2	2.97	4.00 × 10^8^ ± 8.66 × 10^7^	85.7	ND
Intestine	2	5.08	3.00 × 10^8^ ± 5.00 × 10^7^	64.3	10.5 ± 6.8
Intestine	6	4.67	1.67 × 10^8^ ± 5.77 × 10^7^	35.8	15.7 ± 12.1
Colon	6	6.13	3.00 × 10^8^ ± 5.77 × 10^7^	64.3	19.0 ± 4.2
Colon	72	5.28	7.40 × 10^7^ ± 2.51 × 10^7^	15.9	148.3 ± 19.0

CFU = Colony Forming Units, ND = Not determined.

## Data Availability

The original contributions presented in the study are included in the article, further inquiries can be directed to the corresponding authors.

## Abbreviations

The following abbreviations are used in this manuscript:

GABA	Gamma-aminobutyric acid
CNS	Central nervous system
LAB	Lactic acid bacteria
GRAS	Generally Recognized As Safe
GAD	Glutamate decarboxylase
MSG	Monosodium glutamate
RT-qPCR	Real-time reverse transcription quantitative PCR
HPLC	High-performance liquid chromatography
LOD	Limit of detection
LOQ	Limit of quantification
CFU	Colony forming units
PLP	Pyridoxal-5′-phosphate
